# A New Genus of Sap Beetles (Coleoptera: Nitidulidae) from Mid-Cretaceous Amber of Northern Myanmar

**DOI:** 10.3390/insects13100884

**Published:** 2022-09-28

**Authors:** Qian Zhao, Diying Huang, Chenyang Cai

**Affiliations:** 1 State Key Laboratory of Palaeobiology and Stratigraphy, Center for Excellence in Life and Paleoenvironment, Nanjing Institute of Geology and Palaeontology, Chinese Academy of Sciences, Nanjing 210008, China; 2 Nanjing College, University of Chinese Academy of Sciences, Nanjing 211135, China; 3 School of Earth Sciences, University of Bristol, Life Sciences Building, Tyndall Avenue, Bristol BS8 1TQ, UK

**Keywords:** Burmese amber, Nitiduloidea, taxonomy, palaeodiversity, stem group

## Abstract

**Simple Summary:**

In the present paper, we report a new genus and species of sap beetles (family Nitidulidae), *Protonitidula neli* gen. et sp. nov., that was recovered from mid-Cretaceous amber in northern Myanmar, from some 99 million years ago. *Protonitidula neli* possesses morphological characteristics of both Kateretidae and Nitidulidae. Based on the characteristic broad prosternal process, *Protonitidula* is tentatively placed in Nitidulidae. Our discovery enriches the fossil record of the family in the Cretaceous Burmese amber.

**Abstract:**

Nitidulidae is the most diverse family of the recently recognized superfamily Nitiduliodea, but Mesozoic nitidulids that are critical for understanding their early diversification are sparse. Here, we report a new genus and species of Nitidulidae, *Protonitidula neli* gen. et sp. nov., that was recovered from mid-Cretaceous amber in northern Myanmar. The new genus is distinguished from all members of the extant nitidulid subfamilies most prominently by the loose antennal club and the absence of subantennal grooves. *Protonitidula neli* can be excluded from the closely related Kateretidae and classified into Nitidulidae by the broad and apically expanded prosternal process, although it has many pleisiomorphic characters.

## 1. Introduction

Nitidulidae (sap beetles) is the most diverse family of the superfamily Nitiduliodea [1], with there being approximately 350 genera and 4500 species [2]. At present, eleven subfamilies are recognized: Calonecrinae, Maynipeplinae, Epuraeinae, Carpophilinae, Amphicrossinae, Meligethinae, Nitidulinae, Cillaeinae, Cryptarchinae, Cybocephalinae, and Prometopiinae [2,3]. Among them, the morphologically peculiar Cybocephalinae is elevated to the familial level (as Cybocephalidae), and the subfamily Prometopiinae is recognized based on its molecular phylogenetics [3]. However, a recent phylogenomic study clearly shows that Cybocephalinae is nested within Nitidulidae and it should be considered as a subfamily of Nitidulidae, and this suggests that the previous segregation was due to the long-branch attraction in the multigene-based molecular phylogeny [1].

Nitidulidae is widely distributed mainly in the Holarctic and Tropics, except for Maynipeplinae which is in Africa and Calonecrinae which is in Southeast Asia [2]. Extant nitidulids also have a wide range of feeding habits, including mycophagy, phytophagy, inquilinism, and even predation [4]. The key diagnostic features of Nitidulidae are as follows: a subantennal groove that is generally present; an antenna that is usually 11-segmented, with three apical antennomeres forming a club; a mandible that is distinct and curved; an abdomen that is generally sclerotic, with five abdominal ventrites; a pygidium that is exposed; and a tarsal formula that is usually 5-5-5 [2].

The family Kateretidae is a sister to Nitidulidae, and both are members of the recently recognized superfamily Nitiduliodea [1]. The morphological differences between Nitidulidae and Kateretidae are as follows: subantennal grooves that are absent in Kateretidae, but present, generally, in Nitidulidae; procoxal cavities that are open generally in Kateretidae, but close procoxal cavities that occur possibly in Nitidulidae; tibiae that are typically widened posteriorly in Kateretidae, but in Nitidulidae, the protibiae are widened gradually; lateral edges without spinose carinae in Kateretidae, while at least one carinae with rows of spines is present in Nitidulidae [5]. Kateretids are currently important pollinators of angiosperms, as are many species of Nitidulidae. Recent fossil studies have demonstrated that kateretids may have been one of the earliest insects that pollinated early angiosperms, which possibly originated when angiosperms began to flourish during the Cretaceous [6,7,8]. As such, the Mesozoic record of Nitidulidae and Kateretidae from exceptional biotas such as the Burmese amber may provide new insights into the co-evolution of insects and plants.

To date, fossil Nitidulidae are mostly known from the Eocene Baltic amber, with only a few records being from the Cretaceous [9]. Mesozoic nitidulids encompass four species which are classified into two genera, namely *Crepuraea archaica* Kirejtshuk, *Crepuraea explanata* Kirejtshuk, *Crepuraea zherichini* Kirejtshuk and Ponomarenko, *Cyllolithus mirandus* Kirejtshuk, and a nitidulid that is described from Myanmar amber, *Sorodites angustipes* Kirejtshuk [10,11]. Here, we report a new primitive-looking genus and species of nitidulids that has been recovered from the mid-Cretaceous Burmese amber.

## 2. Materials and Methods

The amber piece was ground with sand papers of various sizes and polished with diatomite mud [12]. Photographs were taken using three devices: a Zeiss Stereo Discovery V16 microscope system with incident light and transmitted light, a Zeiss Axio Imager 2 light microscope with a fluorescence imaging system, and a Zeiss LSM 710 confocal laser scanning microscope (CLSM) with digital cameras attached [13,14]. The images were rendered using the Helicon Focus software for Extend depth of field. Figure plates were compiled and arranged in Adobe Photoshop 2021. The type specimen is housed in the Nanjing Institute of Geology and Palaeontology (NIGP), Chinese Academy of Sciences, Nanjing, China.

The Burmese (Kachin) amber that was studied here was derived from an amber mine near the Noije Bum Hill summit site, 20 km southwest of Tanai, in the Hukawng Valley, Kachin Province, northern Myanmar [15,16]. According to palaeontological and radioactive data, the age of the Myanmar amber has been constrained to the middle Cretaceous, Albian (around 98.79 ± 0.62 Ma) [17,18]. The nomenclatural acts that are established, herein, are registered under ZooBank LSID urn:lsid:zoobank.org:pub:D1BD6EC6-693B-41EB-85D8-08CEF04C22AD.

## 3. Systematic Palaeontology

Order: Coleoptera Linnaeus, 1758
Superfamily: Nitiduloidea Latreille, 1802
Family: Nitidulidae Latreille, 1802
Subfamily: unknown
Genus *Protonitidula* gen. nov.
(ZooBank LSID urn:lsid:zoobank.org:act:3EB9530F-00B4-4D4F-83D7-6C6F02794768)
Type species: *Protonitidula neli* sp. nov.

*Diagnosis*: The body is elongated, almost parallel-sided. The semilune mandibles are strongly curved apically. The labrum and clypeus are separated distinctly. The antennal insertions are concealed by protruding expansions of the frons. The antenna has 11 antennomeres, with a loose, 3-segmented antennal club. The antennal grooves are not developed; a jugular process is present. The pronotum is transverse, with a length (horizontally) that is longer than width is, and it has a disc of pronotum that is densely punctated. The elytra are punctated on a disc, with most of the pygidium being exposed. The procoxal cavities are circular and the prosternal process is prolonged behind the procoxae and it is laterally dilated at the apex. There are distinct carinae on the meso- and metatibiae, with rows of spines on the tibiae. The tarsal formula is 5-5-5, with it having simple claws.

*Etymology*: The generic name is a combination of the Latinized prefix *proto*-, meaning giving rise to, and ‘*Nitidula*’, the type genus of Nitidulidae, the family to which the beetle belongs.

*Included species*: Only the type species, *Protonitidula neli* sp. nov.

**Figure 1:**
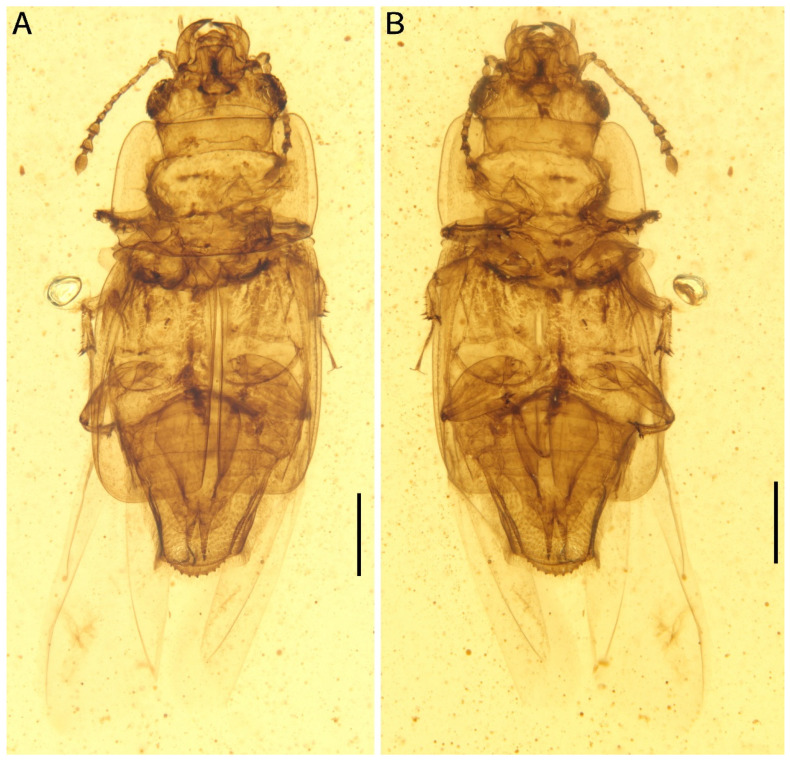
Photomicrographs of *Protonitidula neli* gen. et sp. nov. from mid-Cretaceous Burmese amber under normal reflected light (holotype, NIGP200399). Note that the well-developed hind wings are exposed. (**A**) dorsal view. (**B**) ventral view. Scale bars, 500 μm.

**Figure 2:**
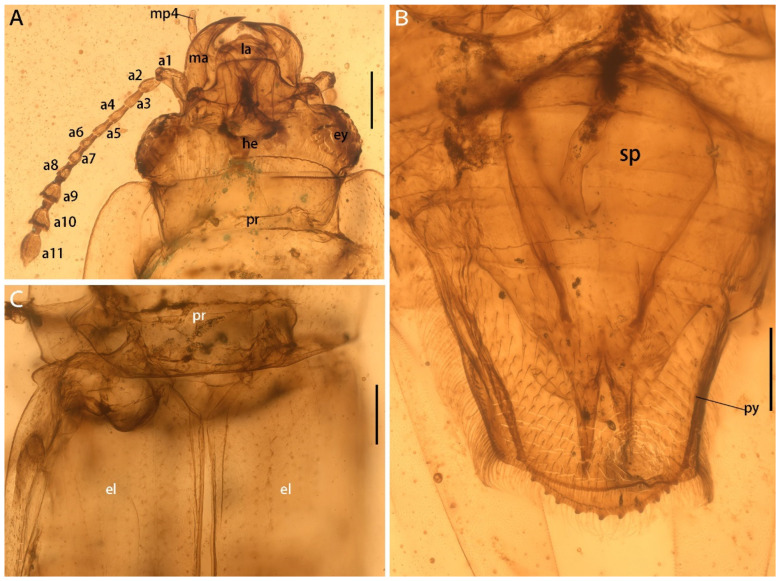
Morphological details of *Protonitidula neli* gen. et sp. nov. from mid-Cretaceous Burmese amber under transmitted light (holotype, NIGP200399). (**A**) Dorsal view of head. (**B**) Pygidium with spermatheca. (**C**) Basal part of elytra. Abbreviations: he, head; ma, mandible; ey, eye; pr, pronotum; mp4, maxillary palpomere 4; la, labrum; el, elytra; a1–11, antennomere 1–11; py, pygidium; sp, spermatheca. Scale bars: 200 μm.

**Figure 3:**
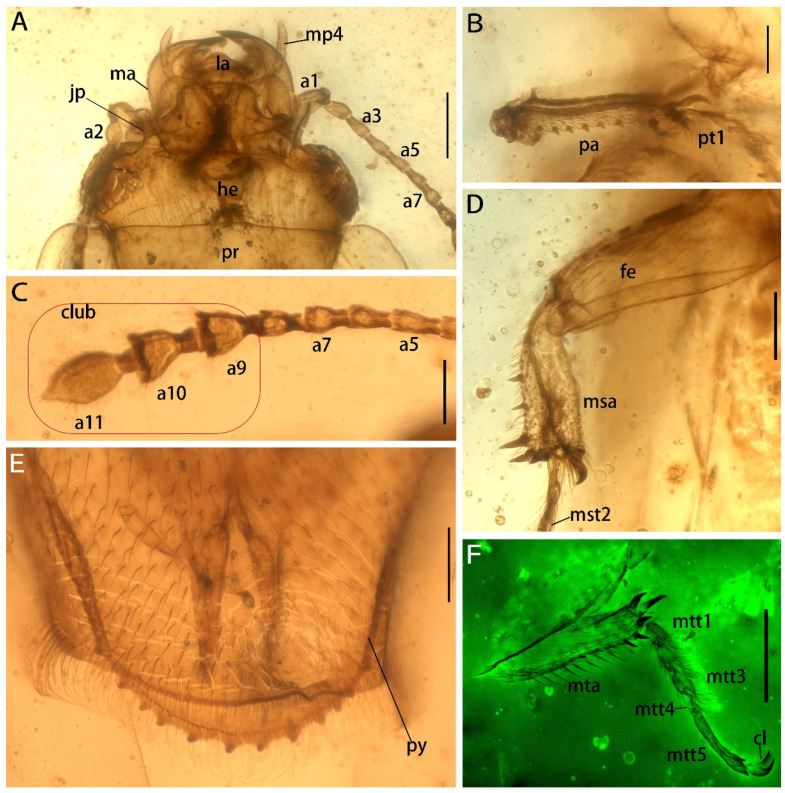
Details of *Protonitidula neli* gen. et sp. nov. from mid-Cretaceous Burmese amber (holotype, NIGP200399) under a confocal laser scanning microscope (CLSM), while others are under transmitted light. (**A**) Ventral view of head. (**B**) Protibia. (**C**) Antenna 5–11. (**D**) Mesotibia. (**E**) Pygidium. (**F**) Metatibia. Abbreviations: jp, jugular process; he, head; ma, mandible; la, labrum; ey, eye; pr, pronotum; mp4, maxillary palpomere 4; a1, antennomere 1; a3, antennomere 3; a5, antennomere 5; a7, antennomere 7; a9, antennomere 9; a10, antennomere 10; a11, antennomere 11; la, labrum; pa, protibial; msa, mesotibia; mta, metatibia; fe, femur; py, pygidium; pt1 protarsomere 1; mst2 mesotarsomere 2; mtt1, metatarsomere 1; mtt3, metatarsomere 3; mtt4, metatarsomere 4; mtt5, metatarsomere 5; cl, claw. Scale bars: 200 μm in A and F, 100 μm in others.

*Protonitidula neli* sp. nov.
(Figure 1, Figure 2 and Figure 3; ZooBank LSID urn:lsid:zoobank.org:act:91582460-1FA1-423F-865A-CD8E282174AD)

*Etymology*: The specific epithet is in honor of Professor Andre Nel, a well-known palaeoentomologist.

*Diagnosis*: The antennal club is 3-segmented, and it is loose; most of pygidium exposed; the procoxal cavities are circular; the prosternal process is broad and expanded apically; there are tibiae with a row of external spines, there are carinae on inner side; the tarsomere 5 is as long as the preceding three segments are combined.

*Holotype*: NIGP200399, Female.

*Type locality and horizon*: Amber mine in the Hukawng Valley, Myitkyina District, Kachin State, Myanmar; Cenomanian to late Albian (mid-Cretaceous) (Mao et al., 2018).

*Description*: The body is enlongated, dorsoventrally flattened, with one exposed abdominal segment from the dorsal view (Figure 1). The body length is 3.4 mm (measured from the mandibular apex to the abdominal apex), and body width is 1.4 mm across the elytra at the broadest point (Figure 1A). The elytra is nearly half of body length (Figure 1). Dense setae are developed on the antennae, legs, and abdomen (Figure 1).

The head is prognathous, and dorsoventrally flattened. The compound eyes are protuberant, enlarged and they are surpassing the temples, ovular, and situated laterally without interfacetal setae (Figure 2A). The frontoclypeal region is protruding anteriorly (Figure 2A). The frons is ladder-shaped and delimited laterally by the dorsal margin of antennal socket; the anterior margin of the frons is straight; the anterolateral margins of the frons is produced over the antennal sockets (Figure 2A). The antennal insertions are moderately separated by approximately three-eighths of the pronotum anterior margin; the occipital suture is distinct (Figure 2A). The temples are poorly developed and short (Figure 2A). The semilune mandibles are extended slightly, with a base width that is twice greater around them than the pedicel length, and the apex is strongly curved such that outer apical margin runs parallel to the anterior margin of the frons, almost (Figure 2A). The maxillary palpi is four-segmented, with an elongated maxillary palpomere 2, a spherical maxillary palpomere 3, and a cylindrical maxillary palpomere 4 (Figure 3A). The antennae are 11-segmented, with a loose antennal club; the scape (antennomere 1) is enlarged; the pedicel (antennomere 2) is 1.5 times wider than the following segment is, with it having an equal length; antennomeres 3–5 are of equal length and width, whereas antennomeres 5–8 become gradually shorter and wider, with the thickest antennomere being 7; antennomeres 9–11 form a loose, non-parallel-sided antennal club, with the antennomeres lengthening apically such that antennomere 11 is 1.8 times longer than antennomere 9 is, with little change in its width; the ultimate antennomere has an acute apex. Subantennal grooves are absent (Figure 2A and Figure 3A,C). The jugal processes is distinct; the right process is 1.2 times longer than left one is; the right process has a sharp, triangular cone shape; the left process is anteriorly truncated, with same length and width (at the broadest point) as the scape (Figure 3A). The prosternal process is wide, expanding beyond the procoxae; the procoxal cavities are circular, and they are separated by an equal length as the procoxal diameter (Figure 1B).

The pronotum transverse is 0.8 mm long and 1.3 mm wide; the anterior margin is straight with a row of setae; the lateral margins are smooth, arc-shaped, and are with thin extensions; the posterior margin is slightly curved; the pronotum is a rectangle, while the corner of the anterior margin is rounder with there being a right angle on the posterior margin; the disc of the pronotum is densely punctated (Figure 1A and Figure 2A). The scutellar shield is small and triangular, and it is one-tenth of elytral length and one-fifth of a single elytron width (Figure 1A).

The elytra is truncated, with one abdominal tergite being exposed; the elytral surface has rows of dense punctures; the elytral carinae are absent; the posterior margin of the elytra is straight in the medial-half and smoothly rounded laterally (Figure 1A and Figure 2C). The hind wings are well developed (Figure 1).

The meso- and metacoxae are moderately separated (Figure 1B). The femora setose is at its widest medially, with a shallow reception for the tibiae (Figure 1B). The tibiae have a row of external spines, with the spines on metatibia being distributed more uniformly; the tibia carinae are on inner side, with there being a strong terminate setae (Figure 3B,D,F). The protibiae are parallel on both sides (Figure 3B). The mesotibiae are dilated apically, where the metatibiae are narrowed apically (Figure 3D,F). The tarsal formula is 5-5-5 (Figure 3B,C); tarsomeres 1–4 are densely setosed ventrally; tarsomere 4 is around half of the size of the former tarsomeres in both length and width; tarsomere 5 is as long as the preceding three segments are combined. The pretarsal claws are simple (Figure 3F).

The abdomen has five ventrites, which are setosed ventrally and pubescent laterally; the ratios of the ventrite lengths along middle line are: 3.25:1.1:1.0:1.5:5.1; ventrite 5 is subtriangular, and apically broadly rounded (Figure 2B). Tergum 5 is lightly longer than ventrite 5, and it is apically serrated (Figure 3E). There is a complete spermatheca in the abdomen, with possible gonocoxities and styli being present (Figure 2B).

## 4. Discussion

The new genus is tentatively attributed to the extant family Nitidulidae based on the following features: the antennae is 11-segmented, with there being a 3-segmented antennal club; most of pygidium is exposed; the procoxal cavities are circular and the prosternal process is prolonged behind the procoxae and it is laterally dilated at the apex; there are distinct carinae on the meso- and metatibiae, with there being rows of spines on the tibiae; and all of the tarsi are 5-segmented. Some of the features of *Protonitidula* are interestingly similar to those of the closely related Kateretidae, including the loose antennal club (which is compact in most extant nitidulids except in many species of the tribe *Mystropini*) and the absence of antennal grooves (which are developed in most nitidulids). These characters are probably ancestral (pleisiomorphic) characters that do not support a close affinity with the more basal clade Kateretidae. More importantly, *Protonitidula* has features that never occur in Kateretidae, but are developed in Nitidulidae, i.e., the prosternal process is expanded behind the procoxae and the distinct carinae are developed on the tibiae [2,5]. In addition, the following features are all found in Nitidulidae: the labrum has minute protuberance in the middle of the anterior margin; the dorsal surface of the elytra is diffusely punctated and pubescent; abdominal ventrite 1 is subequal to ventrites 2–5 combined; and the apex of the pygidium is crenuated [2,19].

Although the overall habitus of *Protonitidula* is closer to that of Nitidulidae, the shape of the antennal club makes it difficult to place *Protonitidula* in any of the existing nitidulid subfamilies. The structure of the putative spermatheca that is observed in the fossils makes us reasonably speculate that this species is female, and that this structure is similar to that of the females in modern Nitidulidae and Kateretidae [19,20]. As such, here we leave *Protonitidula* as *incertae sedis* within Nitidulidae (in the superfamily Nitiduloidea), but we suggest that it may be a basal member of Nitidulidae that may bridge an evolutionary gap between Nitidulidae and Kateretidae.

## Data Availability

All data generated during this study are included in this published article. The type specimen (NIGP200399) is housed in the Nanjing Institute of Geology and Palaeontology, Chinese Academy of Sciences, Nanjing, China.
